# Quantitation of circulating GDF-11 and β2-MG in aged patients with age-related impairment in cognitive function

**DOI:** 10.1042/CS20171028

**Published:** 2017-07-07

**Authors:** Rungong Yang, Shuhong Fu, Liang Zhao, Bei Zhen, Ling Ye, Xiaolu Niu, Xiaoxia Li, Pumin Zhang, Jie Bai

**Affiliations:** 1 Department of Orthopedics, First Affiliated Hospital, Chinese PLA General Hospital, Beijing 100048, China; 2 Department of Clinical Lab of Nanlou, Chinese PLA General Hospital, Beijing 100853, China; 3 State Key Laboratory of Proteomics, Beijing Proteome Research Center, National Center for Protein Sciences (Beijing), Beijing 102206, China; 4 Institute of Geriatrics/Key Laboratory of Normal Aging and Geriatrics, Chinese PLA General Hospital, Beijing 100853, China

**Keywords:** ageing, cognitive impairment, growth differentiation factor 11, β2-microglobulin

## Abstract

Growth differentiation factor 11 (GDF-11) has been implicated in reverse effects of ageing on the central nervous system of humans. β2-microglobulin (β2-MG) has been reported to negatively regulate cognition. However, there is a lot of controversy about the role of GDF-11 and β2-MG in ageing and cognitive regulation. To examine the involvement of GDF-11 and β2-MG in the ageing process and cognitive dysfunction, a total of 51 healthy subjects and 41 elderly patients with different degrees of age-related cognitive impairment participated in the study. We measured plasma GDF-11 and β2-MG levels using ELISA and immunoturbidimetry, respectively. The results were statistically analyzed to evaluate the associations between levels of GDF-11 and β2-MG, and ageing and cognitive impairments. Circulating GDF-11 levels did not decline with age or correlate with ageing in healthy Chinese males. We did not detect differences in circulating GDF-11 levels amongst the healthy advanced age and four cognitive impairment groups. β2-MG levels increased with age, but there was no significant difference between healthy elderly males and advanced age males. Increased levels of β2-MG were observed in the dementia group compared with the healthy advanced age group. Our results suggest that circulating GDF-11 may not exert a protective effect during the ageing process or on cognitive function, and β2-MG may play a role in ageing and cognitive impairment. However, it is possible that the relatively small sample size in the present study affected the quality of the statistical analysis, and future studies are needed to further validate our findings.

## Introduction

Growth differentiation factor 11 (GDF-11) comprises 407 amino acids and is a member of the transforming growth factor β (TGF-β) superfamily that regulates diverse cellular processes [1]. Studies from animal experiments and large human cohorts showed that levels of circulating GDF-11 declined during ageing [2–4]. In heterochronic parabiosis experiments, which can rejuvenate several organs in the older organism of the pair, GDF-11 was identified as a rejuvenation factor [5,6]. Interestingly, injecting recombinant GDF-11 (rGDF-11) into aged animals recapitulates the effects of heterochronic parabiosis [7]. It was also shown that GDF-11 exerts rejuvenating effects on the central nervous system of mice by reversing age-related dysfunction in neuronal stem cells [8]. GDF-11 seems to help the cerebral vasculature and enhance neurogenesis [9]. Currently, a randomized, placebo-controlled, double-blind trial at Stanford University is assessing the safety and efficacy of using plasma from young individuals to treat Alzheimer’s disease [10].

In contrast, a study by Egerman et al. [11] analyzing the function of GDF-11 in young and aged mice found that GDF-11 serum levels increased during ageing, and systemic injection of GDF-11 impaired satellite cell expansion and differentiation, which led to decreased regenerative capacity. Furthermore, an innovative LC–tandem MS (LC-MS/MS) assay performed by Schafer et al. [12] indicated that GDF-11 levels did not decline with age in healthy men. These results were inconsistent with the previous studies described above.

Experiments using heterochronic parabiosis also indicated that circulating pro-ageing factors from the blood of older individuals drove ageing phenotypes in the brain [8,13]. β2-microglobulin (β2-MG), a component of MHC class I (MHC I) molecules that form an active part of the adaptive immune system [14], was identified as a circulating factor that negatively regulates cognitive and regenerative functions in the adult hippocampus in an age-dependent manner [13]. In aged mice, elevated levels of β2-MG were observed in both the plasma and the hippocampus compared with the young mice [15]. Importantly, these findings were validated in banked human plasma and cerebrospinal fluid. Increased systemic levels of soluble β2-MG have been implicated in cognitive impairment associated with chronic hemodialysis [16,17]. Moreover, increased soluble β2-MG has been detected in the cerebral spinal fluid of patients with HIV-associated dementia [18,19] and Alzheimer’s disease [20]. Finally, exogenous β2-MG injected systemically or locally in the hippocampus has been shown to impair hippocampal-dependent cognitive function and neurogenesis in young mice [15].

To further examine the possibility that GDF-11 and β2-MG are involved in the ageing process and cognitive dysfunction, we measured the levels of GDF-11 and β2-MG in human plasma from healthy adult males, healthy elderly males, and elderly males with different degrees of age-related cognitive impairment.

## Materials and methods

### Study population

This was a prospective cohort study. The institutional review board of the Beijing Proteome Research Center State Key Laboratory of Proteomics approved the present study (approval number: SKLP-O201401).

Briefly, between January 2015 and December 2015, we recruited 15 healthy adults, 36 healthy elderly people, and 41 elderly patients with different degrees of age-related cognitive impairment from Chinese PLA General Hospital. Eligible participants met one or more of the following criteria: (i) healthy adults were male, 25–40 years old, and had evidence of healthy medical examinations; (ii) healthy elderly people were male, 60–85 years old, and had evidence of healthy medical examinations and normal cognitive function. Individuals with high blood pressure/diabetes/hyperlipidemia were accepted; (iii) healthy advanced age people were male, >85 years old, and had evidence of healthy medical examinations and normal cognitive function. Individuals with high blood pressure/diabetes/hyperlipidemia were accepted; (iv) elderly patients with age-related cognitive impairments were male, >60 years old, and in a stable general condition. They showed evidence of cognitive impairment by neuropsychological scale assessments that included visuospatial skills, orientation to time and place, executing skills, memory, counting, judgement and understanding, insight, intonation and facial expressions, consciousness, and unresponsiveness. In addition, they had a diagnosis of Alzheimer’s disease or vascular cognitive impairment; and (v) all the participants completed medical examinations that included an interview, a questionnaire, fasting blood tests, urine and stool examinations, an electrocardiogram, an ultrasound, and a chest X-ray or CT scan. We excluded older individuals with congenital cognitive disorders, elevated serum C-reactive protein (>1.0 mg/dl), fever (>37.5°C), impaired liver or kidney function, elevated troponin, or surgery in the previous 2 weeks. The 101 eligible participants were the analytic cohort for the present study, and they provided written informed consents.

Participant demographic characteristics (age, sex, and race), disease history (such as hypertension, diabetes, hyperlipidemia, stroke, and myocardial infarction), as well as history of blood or plasma transfusion and administration of blood products in the previous 1 month were recorded.

### GDF-11, β2-MG, and other biomarker measurements

Venous blood samples were drawn into Vacutainer® Lithium Heparin tubes (BD, Franklin Lakes, NJ, U.S.A.) and centrifuged for 15 min at 2000×***g*** to separate the plasma. The plasma was divided into aliquots and stored at −50°C. Hemolytic or chylous samples were excluded. The concentration of GDF-11 in plasma was determined using the human GDF-11 sandwich ELISA Kit (catalog number: E-EL-H1908c, Elabscience Biotechnology Co., Ltd, Wuhan, China) according to the manufacturer’s instructions. Two parallel measurements were performed for each sample. The quantitation sensitivity of this assay was 9.38 pg/ml, and the intra-assay and inter-assay CVs were <10%.

Concentrations of β2-MG, high-sensitivity C-reactive protein (hs-CRP), and Ig G, A, M, and E as well as light chains in plasma were determined using an immunoturbidimetric analyzer (BN II, Siemens Healthcare Diagnostics Products GmbH, Marburg, Germany). Correlations amongst changes in these measures and cognitive impairments in the elderly were evaluated.

### Statistical analysis

Data were analyzed using the SPSS 17.0 statistical software package. Normality and homogeneity tests of variables were conducted. One-way ANOVA was used for each variable fitting a normal distribution after log transformation, and Dunnett’s test was used for comparison amongst groups of variables with unequal variance. The Kruskal–Wallis rank test was performed for non-normal data in multiple samples, and Mann–Whitney U and Wilcoxon W tests were used for two independent samples. Linear correlation analysis and logistic regression models were used to estimate associations amongst variables. Mean values, S.D., mid-values, and percentiles were calculated using SPSS 17.0. All reported *P*-values are two-tailed with *P*<0.05 set as statistically significant.

## Results

### Demographic and baseline data

A total of 92 male individuals met our inclusion criteria and participated in the present study. Of these, 15 were healthy adults with a mean age of 42.7 years, 12 were healthy elderly people with a mean age of 78.2 years, and 24 were healthy advanced age people with an average age of 90.0 years. Finally, 41 participants were elderly people with cognitive impairments. Based on their clinical diagnosis, we divided the elderly people with cognitive impairments into four subgroups: vascular mild cognitive impairment (MCI) (Va-MCI), vascular dementia (VaD), Alzheimer’s disease cognitive impairment (Ad-MCI), and Alzheimer’s disease dementia (AdD), with 6, 9, 8, and 18 participants per group, respectively. Amongst the 92 individuals, 16 had been administered fresh frozen human plasma, human serum albumin, and/or deproteinated calf blood jelly in the previous month. Differences in age, the three indexes, and medical history amongst the groups were compared by ANOVA. As shown in Table 1, there were significant differences amongst the seven groups in age and the three indexes.

**Table 1:** Comparison of demographics and clinical features amongst the patients in different groups

Variable	Groups							*Χ*^2^
	Healthy (%)			Cognitive impairment (%)				
	Adult	Elderly	Advanced age	Va-MCI	VaD	Ad-MCI	AdD	
Number of subjects	15	12	24	6	9	8	18	
Age (years)	42.7 ± 13.8	78.2 ± 6.4	90.0 ± 2.6	93.7 ± 4.7	91.4 ± 5.4	90.2 ± 4.9	89.44 ± 4.5	32.2^ΔΔ^
Gender								N
Male	15 (100.0)	12 (100.0)	24 (100.0)	6 (100.0)	9 (100.0)	8 (100.0)	18 (100.0)	
Female	0 (0.0)	0 (0.0)	0 (0.0)	0 (0.0)	0 (0.0)	0 (0.0)	0 (0.0)	
Blood/plasma transfusion								8.996
No	15 (100.0)	12 (100.0)	24 (100.0)	5 (83.3)	9 (100.0)	8 (100.0)	16 (88.9)	
Yes	0 (0.0)	0 (0.0)	0 (0.0)	1 (16.7)	0 (0.0)	0 (0.0)	2 (11.1)	
Administered human albumin								8.830
No	15 (100.0)	12 (100.0)	22 (92.0)	4 (66.7)	7 (77.8)	7 (87.5)	14 (77.8)	
Yes	0 (0.0)	0 (0.0)	2 (8.0)	2 (33.3)	2 (22.2)	1 (12.5)	4 (22.2)	
Taking deproteinated calf blood jelly								9.846
No	15 (100.0)	12 (100.0)	24 (100.0)	5 (83.3)	8 (88.9)	8 (100.0)	15 (83.3)	
Yes	0 (0.0)	0 (0.0)	0 (0.0)	1 (16.7)	1 (11.1)	0 (0.0)	3 (16.7)	
Mid-value of detected indexes								
GDF-11 (pg/ml)	25.96	21.40	30.32	101.08	33.95	17.62	23.95	9.668
β2-MG (mg/dl)	0.160	0.255	0.295	0.325	0.450	0.315	0.390	34.661^ΔΔ^

^ΔΔ^, between-group comparison *P*<0.01; N, not determined.

First, the Kolmogorov–Smirnov test indicated that all levels of GDF-11 and β2-MG were not normally distributed (both *P*=0.000). After log transformation, β2-MG levels fitted a normal distribution, but GDF-11 levels did not.

**Figure 1:**
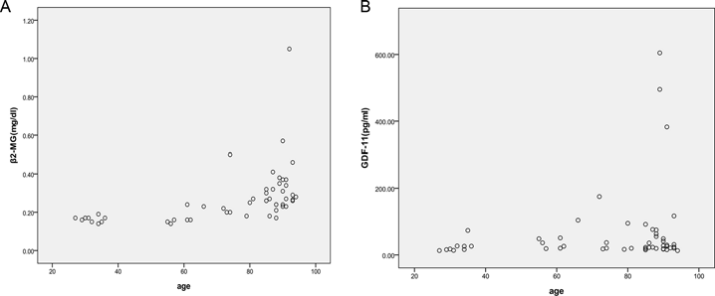
Changes in β2-MG and GDF-11 levels with age in the healthy groups. Although β2-MG levels tended to increase with age (**A**), GDF-11 levels did not depend on age (**B**).

The correlation analysis of two indexes with age in the healthy groups indicated that levels of β2-MG were correlated with age (*P*=0.000); however, GDF-11 levels were not dependent on age (*P*=0.106) (Table 2 and Figure 1).

**Table 2:** Pearson correlation of GDF-11 and β2-MG with age in healthy groups

	β2-MG (mg/dl)	GDF-11 (pg/ml)
*r*-value	0.486^ΔΔ^	0.229
*P*	0.000	0.106

^ΔΔ^, between-group comparison *P*<0.01.

After log transformation, the data from the healthy groups were analyzed by ANOVA. Levels of β2-MG and hs-CRP in the healthy adult group differed significantly from those in the healthy elderly group (*P*=0.000 and 0.018, respectively) and the healthy advanced age group (*P*=0.000 and 0.001, respectively). However, no significant differences were found between the healthy elderly and advanced age groups (*P*=0.256 and 0.702, respectively). For GDF-11, no differences were found amongst the three healthy groups using the Kruskal–Wallis test (*P*=0.127).

**Figure 2:**
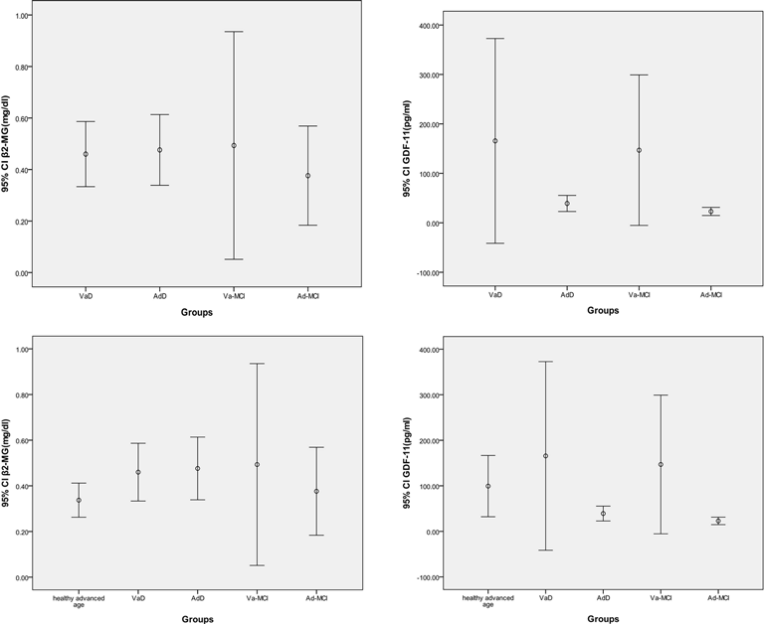
The plasma GDF-11 and β2-MG levels Levels of β2-MG and GDF-11 did not differ amongst the patient groups or between the healthy advanced age group and the four patient groups.

Next, we analyzed the data from the patient groups. There was no difference in age amongst the healthy advanced age group and the four patient groups, Va-MCI, VaD, Ad-MCI, and AdD (*P*=0.274), which eliminated an effect of age on indexes. Levels of β2-MG and GDF-11 did not differ amongst these five groups based on the Kruskal–Wallis test (*P*=0.113 and 0.125, respectively) (Figure 2).

**Figure 3:**
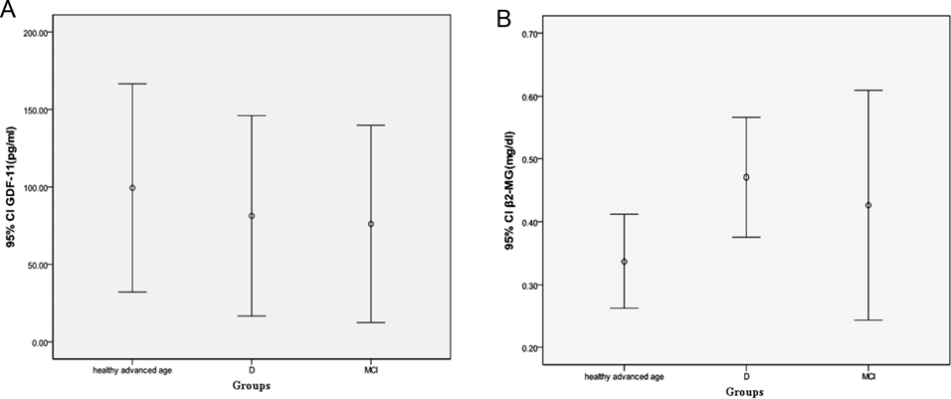
The comparisons of GDF-11 and β2-MG levels between the healthy advanced age group and the D group Levels of β2-MG differed between the healthy advanced age group and the D group after reclassification, but there were no significant differences in GDF-11 levels.

Furthermore, we reclassified all elderly patients into two groups, MCI and dementia (D). We observed that there was still no difference in GDF-11 between the healthy advanced age group and the MCI and D groups (*P*=0.455 and 0.901, respectively). However, we detected a significant difference in β2-MG levels between the healthy advanced age group and the D group at the 0.05 level (*P*=0.027) (Figure 3).

**Figure 4:**
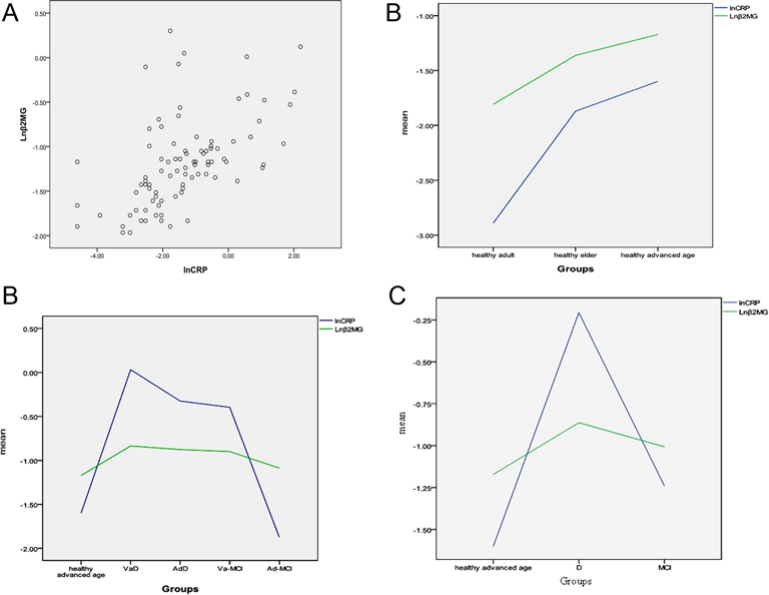
The correlation of β2-MG level with hs-CRP Levels of hs-CRP were closely related to those of β2-MG, and their changes were consistent. (**A**) Pearson correlation of β2-MG with hs-CRP in all participants. (**B**) Consistent changes in β2-MG and hs-CRP in the healthy groups. (**C**) Consistent changes in β2-MG and hs-CRP in the five groups. (**D**) Consistent changes in β2-MG and hs-CRP in the healthy advanced age, D, and MCI groups.

We also analyzed hs-CRP levels in all the participants and found that hs-CRP was related to β2-MG, since their changes were concordant (*r* =0.571, *P*=0.000) (Figure 4A,B). There were significant differences in hs-CRP between the healthy advanced age and D groups (*P*=0.000). In both the healthy and patient groups, β2-MG levels were significantly associated with hs-CRP levels (*r* =0.545, *P*=0.000, and *r* =0.416, *P*=0.001, respectively) (Figure 4C). Upon reclassification of the patient groups into two groups (MCI and D), we found that the correlation between β2-MG and hs-CRP was still present (*r* =0.301, *P*=0.015) (Figure 4D).

**Figure 5:**
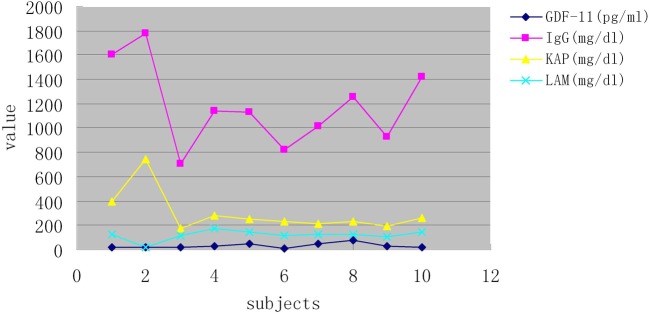
The correlation analysis of GDF-11 and immunoglobulins levels Changes in GDF-11 levels measured in ten subjects were not concordant with immunoglobulins (A, G, M, and E) or light chains, which suggested that the GDF-11 ELISA kits were not affected by immunoglobulins or light chains in plasma.

To assess the specificity of the ELISA kit, we measured the levels of Ig G, A, M, and E as well as light chains in the plasma of ten randomized subjects, including a patient with multiple myeloma accompanying high levels of IgG and κ light chains. We did not detect correlations between GDF-11 levels and immunoglobulins (A, G, M, and E) or light chains, which indicated that GDF-11 measurements were not affected by immunoglobulins or light chains in plasma (Figure 5).

These results suggest that circulating GDF-11 may not exert a protective effect during the ageing process or on cognitive function. However, an elevated β2-MG level may contribute to the ageing process and age-related cognitive impairments. The observation that β2-MG levels were closely related to hs-CRP levels suggests that increased levels of β2-MG may result from various diseases, such as microinflammation, that further promotes the ageing process and age-related cognitive impairments.

## Discussion

Vascular cognitive impairment often results from cerebral ischemia, and Alzheimer’s disease is characterized by loss of neurones. For both the diseases, ageing remains to be the dominant risk factor. Ageing drives cognitive and regenerative impairments in the adult brain, increasing susceptibility to neurodegenerative disorders in healthy individuals [21–24].

Several lines of evidence indicate beneficial effects of GDF-11 on the brain and many other organs. rGDF-11 treatment can partially recapitulate increases in cerebral blood vessel volume, which may ameliorate microvascular ischemic brain disease linked to cognitive decline in the elderly [25]. This effect is due to rGDF-11-induced activation of the TGF-β signaling pathway in brain capillary endothelial cells, which increases their proliferation. rGDF-11 treatment also increases the number of Sox2+ cells in the subventricular zone of aged animals, though not to the extent observed with heterochronic parabiosis [8].

In contrast, Egerman et al. [11] found that GDF-11 serum levels did not decrease but rather increased during ageing. In addition, systemic injection of GDF-11 impaired satellite cell expansion and differentiation, leading to decreased regenerative capacity of aged skeletal muscle [11]. Other researchers also demonstrated that GDF-11 increased during ageing [2]. Several studies [3,11,26] suggested that the monoclonal antibodies and commercial ELISA kits used in previous papers did not discriminate between GDF-11 and myostatin, immunoglobulin, or the IgG light chain, since myostatin (also called GDF-8) is a closely related TGF-β member with an amino acid sequence that is 90% identical with that of GDF-11 [27], and some experiments revealed an affinity of the anti-GDF11/8 antibody (Abcam) for the IgG light chain.

An additional study reported that circulating GDF-11 levels had little physiological relevance, since circulating GDF-11 levels were almost 500 times lower than those of myostatin and could not outcompete myostatin for ActRIIB-binding sites [28]. It was also shown that 74.52% of the variation in GDF-11 levels in different strains of mice was due to the genetic background or strain being used [29]. More recently, a study found that GDF-11 levels increased with age in serum but not in plasma [30].

In the present study, high levels of circulating GDF-11 were detected in some participants with advanced age or dementia, and low levels of circulating GDF-11 were detected in some older subjects with MCI and healthy adults. Plasma transfusions and administration of blood products during the previous 1 month appeared to have little effect on circulating GDF-11 levels despite the relatively few subjects enrolled. The statistical analysis revealed that circulating GDF-11 levels did not decline with age or correlate with ageing in a healthy Chinese population. In addition, we did not detect differences in circulating GDF-11 levels amongst the healthy advanced age, Va-MCI, VaD, Ad-MCI, and AdD groups, or amongst the healthy advanced age, MCI, and D groups. Our results are consistent with those reported by Schafer et al. [12] and Bueno et al. [30]. Together, these findings suggest that circulating GDF-11 is not associated with the causes or severity of cognitive impairment and may not exert protective effects during the ageing process or on cognitive function in humans.

β2-MG is a component of the MHC I complex, which is used by all the cells (except red blood cells) to present intracellular antigens to cytotoxic T cells. Human genome-wide association studies have linked the MHC locus on chromosome 6p21 to degenerative diseases of ageing, which further suggests an active role for these molecules in age-dependent impairments [31]. Elevated levels of β2-MG were observed in both plasma and the hippocampus in aged mice compared with the young mice [15]. These findings were validated in banked human plasma and cerebrospinal fluid. β2-MG has been identified as a potential pro-ageing factor [13] and are found to play a previously unrecognized role in the progression of ageing and age-related impairment of cognitive function [15]. β2-MG-injected young mice resembled aged mice by performing poorly in two cognitive tasks, the radial arm maze and contextual fear conditioning, and they exhibited reduced hippocampal neurogenesis compared with non-injected controls [32]. Furthermore, mice lacking β2-MG were protected against age-related cognitive deficits and maintained normal adult neurogenesis.

In the brain, β2-MG and MHC I can act independent of their canonical immune function to regulate normal brain development, synaptic plasticity, and behavior [33–39]. Results from studies in which isolated hippocampal neural progenitor cells (NPCs) were treated with β2-MG have suggested that β2-MG-induced cognitive deficits may result at least in part from the direct effects of β2-MG on NPCs.

The observation that systemic β2-MG promotes age-related cognitive dysfunction and impairs neurogenesis suggests that β2-MG could be targetted therapeutically in old age. A recent clinical trial in California was the first to test benefits of young blood in older people with Alzheimer’s disease [40].

In our study, levels of β2-MG were correlated with age in a healthy Chinese population. Levels of β2-MG were lower in the healthy adult group than in the healthy elderly and advanced age groups, but there was no significant difference between the healthy elderly and advanced age groups. Moreover, we observed increased levels of β2-MG in the D group compared with the healthy advanced age group. These results suggest that β2-MG may play a role in the ageing process and age-related cognitive impairment. In addition, we analyzed hs-CRP levels in all the participants and found that β2-MG levels were closely related to hs-CRP levels, and their changes were consistent in both the healthy groups and the patient groups. The mechanism underlying these effects is not clear. We hypothesize that, unlike disease biomarkers such as hs-CRP, increased levels of β2-MG may result from various diseases, such as microinflammation, that further promote the ageing process and age-related cognitive impairment.

To the best of our knowledge, no previous study has investigated an association between the ageing process and GDF-11 or β2-MG in the Chinese population. Moreover, little is known regarding the role of GDF-11 or β2-MG in the ageing process and cognitive impairment in the Chinese population. It is possible that the accuracy and specificity of measurements in the current study were not sufficient, and future studies are needed to further clarify the biological activities of GDF-11 and β2-MG in ageing and cognitive impairment.

## Clinical perspectives

- Currently, the effects of GDF-11 and β2-MG on ageing and cognitive impairment remain controversial.
- In the present study, we found that circulating GDF-11 levels did not decline with age or correlate with ageing in healthy Chinese males. We did not detect differences in circulating GDF-11 levels amongst the healthy advanced age and four cognitive impairment advanced age groups. Levels of β2-MG increased with age, but there was no significant difference in β2-MG levels between healthy elderly and advanced age males. Increased levels of β2-MG were observed in the D group compared with the healthy advanced age group.
- These results suggest that circulating GDF-11 may not exert a protective effect during the ageing process or on cognitive function in humans, and that β2-MG may play a role in the ageing process and age-related cognitive impairment.

## Abbreviations

ActRIIB: activin receptor ⅡB
AdD: Alzheimer’s disease dementia
Ad-MCI: Alzheimer’s disease cognitive impairment
CV: coefficient of variation
CT: computed tomography
GDF-11: growth differentiation factor 11
hs-CRP: high-sensitivity C-reactive protein
MCI: mild cognitive impairment
NPC: neural progenitor cell
rGDF-11: recombinant growth differentiation factor 11
TGF-β: transforming growth factor β
Sox2: SRY-related HMG box-containing transcription factor-2
VaD: vascular dementia
Va-MCI: vascular mild cognitive impairment
β2-MG: β2-microglobulin

## References

[1] Nickel J., Sebald W., Groppe J.C. and Mueller T.D. (2009) Intricacies of BMP receptor assembly. Cytokine Growth Factor Rev. 20, 367–377 19926516 10.1016/j.cytogfr.2009.10.022

[2] Brun C.E. and Rudnicki M.A. (2015) GDF11 and the mythical fountain of youth. Cell Metab. 22, 54–56 26003784 10.1016/j.cmet.2015.05.009

[3] Poggioli T., Vujic A., Yang P., Macias-Trevino C., Uygur A., Loffredo F.S. (2016) Circulating growth differentiation factor 11/8 levels decline with age. Circ. Res. 118, 29–37 26489925 10.1161/CIRCRESAHA.115.307521 PMC4748736

[4] Olson K.A., Beatty A.L., Heidecker B., Regan M.C., Brody E.N., Foreman T. (2015) Association of growth differentiation factor 11/8, putative anti-ageing factor, with cardiovascular outcomes and overall mortality in humans: analysis of the heart and soul and hunt3 cohorts. Eur. Heart J. 36, 3426–3434 26294790 10.1093/eurheartj/ehv385 PMC4685178

[5] Conboy I.M., Conboy M.J., Wagers A.J., Girma E.R., Weissman I.L. and Rando T.A. (2005) Rejuvenation of aged progenitor cells by exposure to a young systemic environment. Nature 433, 760–764 15716955 10.1038/nature03260

[6] Sinha M., Jang Y.C., Oh J., Khong D., Wu E.Y., Manohar R. (2014) Restoring systemic GDF11 levels reverses age-related dysfunction in mouse skeletal muscle. Science 344, 649–652 24797481 10.1126/science.1251152 PMC4104429

[7] Loffredo F.S., Steinhauser M.L., Jay S.M., Gannon J., Pancoast J.R., Yalamanchi P. (2013) Growth differentiation factor 11 is a circulating factor that reverses age-related cardiac hypertrophy. Cell 153, 828–839 23663781 10.1016/j.cell.2013.04.015 PMC3677132

[8] Katsimpardi L., Litterman N.K., Schein P.A., Miller C.M., Loffredo F.S., Wojtkiewicz G.R. (2014) Vascular and neurogenic rejuvenation of the aging mouse brain by young systemic factors. Science 344, 630–634 24797482 10.1126/science.1251141 PMC4123747

[9] Wagers A.J. and Conboy I.M. (2005) Cellular and molecular signatures of muscle regeneration: current concepts and controversies in adult myogenesis. Cell 122, 659–667 16143100 10.1016/j.cell.2005.08.021

[10] Villeda S.A., Plambeck K.E., Middeldorp J., Castellano J.M., Mosher K.I., Luo J. (2014) Young blood reverses age-related impairments in cognitive function and synaptic plasticity in mice. Nat. Med. 20, 659–663 24793238 10.1038/nm.3569 PMC4224436

[11] Egerman M.A., Cadena S.M., Gilbert J.A., Meyer A., Nelson H.N., Swalley S.E. (2015) GDF11 increases with age and inhibits skeletal muscle regeneration. Cell Metab. 22, 164–174 26001423 10.1016/j.cmet.2015.05.010 PMC4497834

[12] Schafer M.J., Atkinson E.J., Vanderboom P.M., Kotajarvi B., White T.A., Moore M.M. (2016) Quantification of GDF11 and myostatin in human aging and cardiovascular disease. Cell Metab. 23, 1207–1215 27304512 10.1016/j.cmet.2016.05.023 PMC4913514

[13] Villeda S.A., Luo J., Mosher K.I., Zou B., Britschgi M., Bieri G. (2011) The ageing systemic milieu negatively regulates neurogenesis and cognitive function. Nature 477, 90–94 21886162 10.1038/nature10357 PMC3170097

[14] Zijlstra M., Bix M., Simister N.E., Loring J.M., Raulet D.H. and Jaenisch R. (1990) β2-Microglobulin deficient mice lack CD4^−^8^+^cytolytic T cells. Nature 344, 742–746 2139497 10.1038/344742a0

[15] Smith L.K., He Y., Park J.-S., Bieri G., Snethlage C.E., Lin K. (2015) β2-microglobulin is a systemic pro-aging factor that impairs cognitive function and neurogenesis. Nat. Med. 21, 932–937 26147761 10.1038/nm.3898 PMC4529371

[16] Murray A.M. (2008) Cognitive impairment in the aging dialysis and chronic kidney disease populations: an occult burden. Adv. Chronic Kidney Dis. 15, 123–132 18334236 10.1053/j.ackd.2008.01.010 PMC2504691

[17] Corlin D.B., Sen J.W., Ladefoged S., Lund G.B., Nissen M.H. and Heegaard N.H. (2005) Quantification of cleaved β2-microglobulin in serum from patients undergoing chronic hemodialysis. Clin. Chem. 51, 1177–1184 15890888 10.1373/clinchem.2005.049544

[18] McArthur J.C., Nance-Sproson T.E., Griffin D.E., Hoover D., Selnes O.A., Miller E.N. (1992) The diagnostic utility of elevation in cerebrospinal fluid β2-microglobulin in HIV-1 dementia. Multicenter AIDS Cohort Study. Neurology 42, 1707–1712 1355286 10.1212/wnl.42.9.1707

[19] Brew B.J., Dunbar N., Pemberton L. and Kaldor J. (1996) Predictive markers of AIDS dementia complex: CD4 cell count and cerebrospinal fluid concentrations of β2-microglobulin and neopterin. J. Infect. Dis. 174, 294–298 8699058 10.1093/infdis/174.2.294

[20] Carrette O., Demalte I., Scherl A., Yalkinoglu O., Corthals G., Burkhard P. (2003) A panel of cerebrospinal fluid potential biomarkers for the diagnosis of Alzheimer’s disease. Proteomics 3, 1486–1494 12923774 10.1002/pmic.200300470

[21] Hedden T. and Gabrieli J.D. (2004) Insights into the ageing mind: a view from cognitive neuroscience. Nat. Rev. Neurosci. 5, 87–96 14735112 10.1038/nrn1323

[22] Mattson M.P. and Magnus T. (2006) Ageing and neuronal vulnerability. Nat. Rev. Neurosci. 7, 278–294 16552414 10.1038/nrn1886 PMC3710114

[23] Small S.A., Schobel S.A., Buxton R.B., Witter M.P. and Barnes C.A. (2011) A pathophysiological framework of hippocampal dysfunction in ageing and disease. Nat. Rev. Neurosci. 12, 585–601 21897434 10.1038/nrn3085 PMC3312472

[24] Rao M.S., Hattiangady B. and Shetty A.K. (2006) The window and mechanisms of major age-related decline in the production of new neurons within the dentate gyrus of the hippocampus. Aging Cell 5, 545–558 17129216 10.1111/j.1474-9726.2006.00243.x

[25] Selnes O.A. and Vinters H.V. (2006) Vascular cognitive impairment. Nat. Clin. Pract. Neurol. 2, 538–547 16990827 10.1038/ncpneuro0294

[26] Smith S.C., Zhang X., Zhang X., Gross P., Starosta T., Mohsin S. (2015) GDF11 does not rescue aging-related pathological hypertrophy. Circ. Res. 117, 926–932 26383970 10.1161/CIRCRESAHA.115.307527 PMC4636963

[27] Dschietzig T.B. (2014) Myostatin - from the mighty mouse to cardiovascular disease and cachexia. Clin. Chim. Acta 433, 216–224 24680839 10.1016/j.cca.2014.03.021

[28] Rodgers B.D. and Eldridge J.A. (2015) Reduced circulating GDF11 is unlikely responsible for age-dependent changes in mouse heart, muscle, and brain. Endocrinology 156, 3885–3888 26372181 10.1210/en.2015-1628

[29] Zhou Y., Jiang Z., Harris E.C., Reeves J., Chen X. and Pazdro R. (2016) Circulating concentrations of growth differentiation factor 11 are heritable and correlate with life span. J. Gerontol. A Biol. Sci. Med. Sci. 71, 1560–1563 26774117 10.1093/gerona/glv308

[30] Bueno J.L., Ynigo M., de Miguel C., Gonzalo-Daganzo R.M., Richart A., Vilches C. (2016) Growth differentiation factor 11 (GDF11) - A promising anti-ageing factor - is highly concentrated in platelets. Vox Sang. 111, 434–436 27509407 10.1111/vox.12438

[31] Jeck W.R., Siebold A.P. and Sharpless N.E. (2012) Review: a meta-analysis of GWAS and age-associated diseases. Aging Cell 11, 727–731 22888763 10.1111/j.1474-9726.2012.00871.x PMC3444649

[32] Filiano A.J. and Kipnis J. (2015) Breaking bad blood: β2-microglobulin as a pro-aging factor in blood. Nat. Med. 21, 844–845 26248266 10.1038/nm.3926

[33] Lee H., Brott B.K., Kirkby L.A., Adelson J.D., Cheng S., Feller M.B. (2014) Synapse elimination and learning rules coregulated by MHC class I H2-Db. Nature 509, 195–200 24695230 10.1038/nature13154 PMC4016165

[34] Loconto J., Papes F., Chang E., Stowers L., Jones E.P., Takada T. (2003) Functional expression of murine V2R pheromone receptors involves selective association with the M10 and M1 families of MHC class Ib molecules. Cell 112, 607–618 12628182 10.1016/s0092-8674(03)00153-3

[35] Boulanger L.M. and Shatz C.J. (2004) Immune signaling in neural development, synaptic plasticity and disease. Nat. Rev. Neurosci. 5, 521–531 15208694 10.1038/nrn1428

[36] Shatz C.J. (2009) MHC class I: an unexpected role in neuronal plasticity. Neuron 64, 40–45 19840547 10.1016/j.neuron.2009.09.044 PMC2773547

[37] Huh G.S., Boulanger L.M., Du H., Riquelme P.A., Brotz T.M. and Shatz C.J. (2000) Functional requirement for class I MHC in CNS development and plasticity. Science 290, 2155–2159 11118151 10.1126/science.290.5499.2155 PMC2175035

[38] Goddard C.A., Butts D.A. and Shatz C.J. (2007) Regulation of CNS synapses by neuronal MHC class I. Proc. Natl Acad. Sci. U.S.A. 104, 6828–6833 17420446 10.1073/pnas.0702023104 PMC1871870

[39] Glynn M.W., Elmer B.M., Garay P.A., Liu X.B., Needleman L.A., Ei-Sabeawy F. (2011) MHCI negatively regulates synapse density during the establishment of cortical connections. Nat. Neurosci. 14, 442–451 21358642 10.1038/nn.2764 PMC3251641

[40] Scudellari M. (2015) Blood to blood. Nature 157, 426–429 10.1038/517426a 25612035

